# Enteric Coronavirus in Ferrets, the Netherlands

**DOI:** 10.3201/eid1708.110115

**Published:** 2011-08

**Authors:** Lisette B.V. Provacia, Saskia L. Smits, Byron E. Martina, V. Stalin Raj, Petra v.d. Doel, Geert v. Amerongen, Hanneke Moorman-Roest, Albert D.M.E. Osterhaus, Bart L. Haagmans

**Affiliations:** Author affiliations: Erasmus Medical Center, Rotterdam, the Netherlands (L.B.V. Provacia, S.L. Smits, B.E. Martina, V.S. Raj, P. v.d. Doel, G. v. Amerongen, A.D.M.E. Osterhaus, B.L. Haagmans);; Ferret Clinic Brouwhuis, Helmond, the Netherlands (H. Moorman-Roest)

**Keywords:** coronavirus, ferret, eneteric, viruses, the Netherlands, letter, *Suggested citation for this article:* Provacia LBV, Smits SL, Martina BE, Raj VS, v.d. Doel P, v. Amerongen G, et al. Enteric coronavirus in ferrets, the Netherlands [letter]. Emerg Infect Dis [serial on the Internet]. 2011 Aug [*date cited*]. http://dx.doi.org/10.3201/eid1708.110115

**To the Editor:** Coronaviruses (CoVs) are enveloped, positive-sense, single-stranded RNA viruses that can cause acute and chronic respiratory, enteric, and central nervous system disease in a variety of animal species (*1*). Recently, a novel ferret enteric CoV (FRECV) was indentified in domesticated ferrets (*Mustela putorius*) in which epizootic catarrhal enteritis had been diagnosed; the illness was characterized by foul-smelling green diarrhea with high mucus content, lethargy, anorexia, and vomiting (*2*). Another ferret CoV emerged in ferrets for which systemic pyogranulomatous inflammation, resembling the clinical and pathologic features of feline infectious peritonitis (FIP), was diagnosed (*3**–**5*).

In 2010, we investigated the prevalence of CoV antibodies in 85 asymptomatic ferrets obtained from 1 ferret farm in the Netherlands. Previous studies have shown that antibodies against different members of the α-CoVs show broad cross-reactivity (*6*). We used FIP virus (FIPV)–infected cells to screen for CoV antibodies in an indirect immunoperoxidase assay. Because 32% of the ferret serum had a titer >20 in this assay, we concluded that these animals most likely had been exposed to a CoV. To test for a CoV in these animals, we analyzed RNA extracted from rectal swabs with a degenerate set of primers to amplify a conserved region within open reading frame (ORF) 1 of CoVs (*7*). Remarkably, 36 (42%) of samples tested were PCR positive, suggesting excretion of a CoV by a substantial proportion of ferrets tested. To corroborate that the CoV detected in the rectal swabs was a ferret CoV (FRCoV), we amplified and sequenced the nucleocapsid protein by using primer pair 5′-TCCCCGCGGGGCTGGCAACGGACAACGTGT-3′ and 5′-CCCAAGCTTTTAGTTTAGTTGACTAATAATTTCA-3′. Phylogenetic analyses of 2 of the sequences obtained indicated a variant nucleocapsid that was similar to other FRCoVs described previously but that did not group with 1 of these sequences directly (Figure). Amino acid alignment of 1 of these sequences (FRCoV-511c) with FRECV-MSU2 demonstrated 91.8% identity and 95.7% similarity, whereas this virus shows 89.3% identity and 95.2% similarity to ferret systemic CoV (FRSCV-MSU1).

**Figure:**
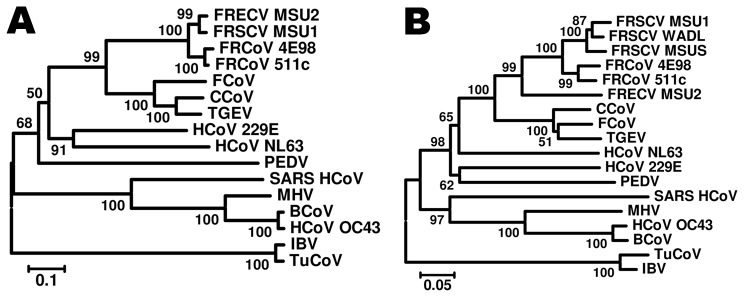
Phylogenetic tree based on nucleotide sequences of the nucleocapsid (A) and spike gene (B) of ferret coronaviruses (FRCoVs) 4E98 (GenBank accession nos. JF260916 and JF260914, respectively) and 511c (accession nos. JF260915 and JF260913, respectively) and other coronaviruses (CoVs). Partial nucleotide sequences were aligned by using ClustalX (www.clustal.org) and a neighbor-joining Kimura 2-parameter model with 1,000 bootstrap replicates; avian CoVs were used as outgroup sequences (p-distance; allowing gaps or missing data). Other CoVs shown (abbreviation, GenBank accession number): ferret coronavirus (FRECV MSU2, GU338457); ferret systemic coronavirus (FRSCV MSU1, GU338456); feline coronavirus (FCoV, DQ010921); canine coronavirus (CCoV, AY342160); transmissible gastroenteritis virus (TGEV, AF104420); human coronavirus (HCoV NL63, DQ445911); porcine epidemic diarrhea virus (PEDV, AF353511); severe acute respiratory syndrome coronavirus (SARS-HCoV, NC_004718); murine hepatitis virus (MHV, AY700211); bovine coronavirus (BCoV, U00735); infectious bronchitis virus (IBV, AY363968); and turkey coronavirus (TuCov, AF111997). Scale bars indicate nucleotide substitutions per site.

On the basis of obtained and published nucleocapsid sequences (*2**,**4*), we developed a TaqMan reverse transcription PCR to detect viral RNA using the following primers and degenerate probe: forward, 5′-TTGGAAAGAATGGTGCTAAAACTG-3′; reverse, 5′-CATTAGGCACGTTACCATCAAATT-3′; and probe, 5′-TAGGAACRCGTGGCACCAACCAA-3′. Using this more specific and sensitive assay, we detected viral RNA in 63% of the rectal swabs tested; other CoVs including FIPV, severe acute respiratory syndrome–CoV, and human CoV NL-63, were not amplified by this assay (data not shown). All samples that had tested positive in the ORF1-CoV PCR were confirmed positive with this TaqMan assay. To analyze FRCoV in ferrets from geographically distinct sites, we tested fecal samples from 90 animals without signs of disease (including epizootic catarrhal enteritis) from 39 different locations in the Netherlands. FRCoV nucleocapsid TaqMan and ORF1-CoV PCR demonstrated that 61% of the fecal samples and 72% of the locations were positive. Multiple testing of fecal swabs at different times and use of FRCoV-specific antibody assays would probably further increase the FRCoV prevalence rate.

Further partial sequence analysis of the spike gene by using primers 5′-AARRTTAATGAGTGTGTGMGDTCA-3′ and 5′- CAACTCTYTTAAGCCARTCAAGG-3′ clearly showed that these viruses are more closely related to systemic FRCoVs than to FRECV (Figure). Amino acid alignment of 1 of these sequences (FRCoV-511c) with FRECV-MSU2 demonstrated 78% identity and 89% similarity, and FRCoV-511c shows 86% identity and 92% similarity to FRSCV-MSU1.

After identification of severe acute respiratory syndrome CoV in humans in 2003 and related viruses in civet cats and bats, an increase in CoV surveillance in different animal species resulted in identification and characterization of a broad range of previously unrecognized CoVs (*8*). Here we report an enteric FRCoV circulating in the Netherlands in a high percentage of asymptomatic ferrets. The ferrets tested did not have a previous record of foul-smelling green diarrhea described previously to be associated with FRECV, a virus detected in the United States and further characterized in 2006 (*2*). On the basis of the phylogenetic analysis of the spike sequences, FRECV-MSU2 might have evolved through recombination with some other unknown CoV. Alternatively, the viruses isolated in the Netherlands grouped more closely with FRCoVs causing systemic disease (e.g., FRSCV-MSU1). Thus far, no evidence indicates that the animals testing positive for the enteric CoV showed clinical disease that pointed to pyogranulomatous inflammation, necrosis with or without perivasculitis, and vasculitis in abdominal and visceral organs associated with the systemic variant. Further genetic characterization of these enteric FRCoVs variants might show genetic differences that could explain the apparent pathotypes of these 2 FRCoVs. FRCoVs might evolve through mutation or deletion into viruses that cause systemic disease, or alternatively, different FRCoVs are circulating, analogous to the hypotheses put forward to explain the occurrence of FIP (*9**,**10*). Given the use of ferrets in testing efficacy of influenza virus vaccines and the propensity of CoVs to cross species barriers, further surveillance and investigation of the biology of these emerging FRCoVs is warranted.

## References

[1] Weiss SR, Navas-Martin S. Coronavirus pathogenesis and the emerging pathogen severe acute respiratory syndrome coronavirus. Microbiol Mol Biol Rev. 2005;69:635–64. 10.1128/MMBR.69.4.635-664.2005 16339739 PMC1306801

[2] Wise AG, Kiupel M, Maes RK. Molecular characterization of a novel coronavirus associated with epizootic catarrhal enteritis (ECE) in ferrets. Virology. 2006;349:164–74. 10.1016/j.virol.2006.01.031 16499943 PMC7111814

[3] Garner MM, Ramsell K, Morera N, Juan-Salles C, Jimenez J, Ardiaca M, Clinicopathologic features of a systemic coronavirus-associated disease resembling feline infectious peritonitis in the domestic ferret (*Mustela putorius*). Vet Pathol. 2008;45:236–46. 10.1354/vp.45-2-236 18424841

[4] Wise AG, Kiupel M, Garner MM, Clark AK, Maes RK. Comparative sequence analysis of the distal one-third of the genomes of a systemic and an enteric ferret coronavirus. Virus Res. 2010;149:42–50. 10.1016/j.virusres.2009.12.011 20079778 PMC7114374

[5] Martínez J, Reinacher M, Perpinan D, Ramis A. Identification of group 1 coronavirus antigen in multisystemic granulomatous lesions in ferrets (*Mustela putorius furo*). J Comp Pathol. 2008;138:54–8. 10.1016/j.jcpa.2007.10.002 18067916 PMC7094249

[6] Vlasova AN, Zhang X, Hasoksuz M, Nagesha HS, Haynes LM, Fang Y, Two-way antigenic cross-reactivity between severe acute respiratory syndrome coronavirus (SARS-CoV) and group 1 animal CoVs is mediated through an antigenic site in the N-terminal region of the SARS-CoV nucleoprotein. J Virol. 2007;81:13365–77. 10.1128/JVI.01169-07 17913799 PMC2168854

[7] Ksiazek TG, Erdman D, Goldsmith CS, Zaki SR, Peret T, Emery S, A novel coronavirus associated with severe acute respiratory syndrome. N Engl J Med. 2003;348:1953–66. 10.1056/NEJMoa030781 12690092

[8] Woo PC, Lau SK, Huang Y, Yuen KY. Coronavirus diversity, phylogeny and interspecies jumping. Exp Biol Med (Maywood). 2009;234:1117–27. 10.3181/0903-MR-94 19546349

[9] Vennema H, Poland A, Foley J, Pedersen NC. Feline infectious peritonitis viruses arise by mutation from endemic feline enteric coronaviruses. Virology. 1998;243:150–7. 10.1006/viro.1998.9045 9527924 PMC7131759

[10] Brown MA, Troyer JL, Pecon-Slattery J, Roelke ME, O’Brien SJ. Genetics and pathogenesis of feline infectious peritonitis virus. Emerg Infect Dis. 2009;15:1445–52. 10.3201/eid1509.081573 19788813 PMC2819880

